# Case of Purpura Hemorrhagica Resulting in Dropsy

**Published:** 1880-09

**Authors:** Dudley M. Culver

**Affiliations:** Whitesville, Indiana


ARTICLE IV.

### CASE OF PURPURA HEMORRHAGICA RESULTING IN DROPSY.

BY DUDLEY M. CULVER, M. D., WHITESVILLE, INDIANA.

I was called, July 7th, to see Frank R, aged 7 years, had been feeble for several days, was having frequent discharges of blood from his bowels, which had a cadaveric oder, had an effusionof blood un der the skin in places, and as the effusion developed it was at first purple, then bright red, then a muddy color, and finally copper colored. Such was the condition the skin was in; and when the effusion left one part of the body, it would manifest itself in another; finally, a few sores were found on the body, and the cervical and Inguinal Glands swelled up and became very painful, all this with the frequent bloody discharges from his bowels, amaciated my patient very rapidly. There was odema of the feet, extreme tenderness of the muscles of the leg, pain in abdomen, but no tympanites. The ears were ecchymosed untill they bore no resemblance to ears. In one week I fortunately had stopped this effusion and the patient seemed to be doing well. Appetite returned, but to my chagrin and sad disappointment, acute Albuminuria set in, resulting in general Dropsy, and finally suppression of the urine ensued. I tried every Diuretic and Diaphoretic singly and combined with but little effect. He died July 26th, after having tetanic spasms for six hours. There was dropsical effusion involving the lungs and the heart. Fortunately, such cases are met with but seldom; I never having met with one before, and I give it to the readers of your valuable Journal simply as a matter of record; certainly with no view to specific treatment, and I hope that should any of your readers have treated such cases with better success, they will report them for the benefit of the profession, for nothing is more consoling to the busy Physician than to meet with accounts of just such cases in is Medical Journals, as he may be treating at the time. Glad, indeed, would I have been whilst treating this case to have seen in my medical journal the description and successful treatment of just such an one. Let us hear from you, brethren, through the pages of the *Independent Practitioner*.

